# Wild Relatives of the Eggplant (*Solanum melongena* L.: Solanaceae): New Understanding of Species Names in a Complex Group

**DOI:** 10.1371/journal.pone.0057039

**Published:** 2013-02-22

**Authors:** Sandra Knapp, Maria S. Vorontsova, Jaime Prohens

**Affiliations:** 1 Department of Life Sciences, Natural History Museum, Cromwell Road, London, United Kingdom; 2 Herbarium, Library, Art and Archives, Royal Botanic Gardens Kew, Richmond, Surrey, United Kingdom; 3 Instituto de Conservación y Mejora de la Agrodiversidad Valenciana, Universitat Politècnica de València, Valencia, Spain; University College London, United Kingdom

## Abstract

**Background:**

The common or brinjal eggplant (*Solanum melongena* L.) belongs to the Leptostemonum Clade (the “spiny” solanums) of the species-rich genus *Solanum* (Solanaceae). Unlike most of the genus, the eggplant and its relatives are from the Old World; most eggplant wild relatives are from Africa. An informal system for naming eggplant wild relatives largely based on crossing and other biosystematics data has been in use for approximately a decade. This system recognises several forms of two broadly conceived species, *S. incanum* L. and *S. melongena*. Recent morphological and molecular work has shown that species-level differences exist between these entities, and a new species-level nomenclature has been identified as necessary for plant breeders and for the maintenance of accurately named germplasm.

**Methodology/Principal Findings:**

We examined herbarium specimens from throughout the wild species ranges as part of a larger revision of the spiny solanums of Africa. Based on these morphological and molecular studies, we delimited species in the group to which the common eggplant belongs and constructed identification keys for the group. We also examined the monophyly of the group considered as the eggplant relatives by previous authors.

**Conclusions/Significance:**

We recognise ten species in this group: *S. aureitomentosum* Bitter, *S. campylacanthum* A.Rich., *S. cerasiferum* Dunal, *S. incanum* L., *S. insanum* L., *S. lichtensteinii* Willd., *S. linnaeanum* Hepper & P.-M.L.Jaeger, *S. melongena* L., *S. rigidum* Lam. and S. *umtuma* Voronts. & S.Knapp. We review the history of naming and provide keys and character lists for all species. Ploidy level differences have not been investigated in the eggplant wild relatives; we identify this as a priority for improvement of crop wild relative use in breeding. The application of species-level names to these entities will help focus new collecting efforts for brinjal eggplant improvement and help facilitate information exchange.

## Introduction

Eggplants (or aubergines, *Solanum melongena* L.) are the second most important solanaceous fruit crop after tomato (*S. lycopersicum* L.). Both are members of the large and species-rich genus *Solanum* L. (Solanaceae), as is the potato (*S. tuberosum* L.). *Solanum* is one of the ten most species-rich genera of flowering plants [1] and has approximately 1400 species that occur on all continents except Antarctica in a wide variety of habitats from deserts to mountain slopes high above treeline. Species-level taxonomy of such large groups is always challenging, but with the NSF-funded Planetary Biodiversity Inventory project PBI Solanum, a monograph at the species-level of the entire group is becoming a reality (see Solanaceae Source, http://www.solanaceaesource.org; [2]). The genus can be divided into 13 major clades, the largest of which comprises the spiny solanums, the Leptostemonum clade (subgenus *Leptostemonum* Bitter), with ca. 450 species distributed worldwide.

Comparison of the genetics of solanaceous crop species is facilitated by the high degree of synteny across the family [3]. The recent sequencing of reference genomes for potato [4] and tomato [5] has unlocked a wealth of information about the domestication process and the genetic control of characteristics important for human use such as tuberisation in potato and fruit quality in tomato. Potato and tomato are both members of the Potato clade of *Solanum* [6] that comprises some 200 species that are exclusively New World in distribution. Species-level circumscription and relationships in this group have largely been resolved [7], [8], [9], [10], [11]; this has facilitated other biological analyses [12], [13], [14] that depend on species-level identities and knowledge of species relationships.

Interest in the wild relatives of agronomically important crops (crop wild relatives or CWR [15]) for breeding in the face of environmental change means that a species-level understanding of the identity and relationships of these taxa is of more than marginal interest. Judging what species were of interest as CWR has traditionally followed the gene-pool concept, first articulated by Harlan and de Wet [16]. They suggested that CWR could be arranged as a set of nested sets, with the crop and its wild progenitor (in their scheme the landraces plus wild and weedy forms of the crop identified as subspecies) at the centre, and increasingly less crossable taxa in larger inclusive sets. Maxted et al. [17] adapted the concept so that CWR could still be identified in the absence of crossing and genetic diversity information by using the existing taxonomic hierarchy; this taxon-group concept definition of CWR used relationships, whether assessed using explicitly phylogenetic methods or more traditional morphological similarity assessments, as the criteria for constructing a nested set of relationships analogous to the gene-pool concept. In large genera like *Solanum*, both these concepts are challenging, firstly because crossability is often not directly related to relatedness, secondly because crossability relationships are difficult to work out with so many taxa to test, and thirdly because a genus with so many species in unworkable as a category; taxon-group 4 of [17] is the genus.

Eggplants, of which there are three cultivated species only one of which we are concerned with here (see below), are members of the large and taxonomically challenging Leptostemonum clade [18]. The three cultivated eggplants are all Old World in origin; the gboma eggplant *Solanum macrocarpon* L. and the scarlet eggplant *S. aethiopicum* L. are mainly grown locally in Africa but are also cultivated elsewhere as minor crops [19] while the brinjal or common eggplant (also known as aubergine) *S. melongena* L. is grown worldwide. *Solanum melongena* was identified by Vavilov [20] as typical of the “Indo-Chinese centre of origin”. Both Indian and Chinese domestication origins have been postulated, and there is some evidence of a possible third domestication event in Indonesia/Malaysia (summarized in [19]). Recent views, however, are converging on a minimum of two separate domestication events [19], [21] in what is now India and China. Identification of domestication origins are used to identify areas for collection of high genetic diversity for crop improvement [22]. Although all three species of eggplant are partially interfertile [23], the two African species are not thought to be closely related to *S. melongena* [24]. Their relationships and origins are discussed in [25] and further discussion of eggplants in this paper refers to *S. melongena* only.

The taxonomy of the Old World members of the Leptostemonum clade has been problematic for more than a century. Morphological similarity between Old and New World species led some authors [24], [26] to postulate multiple introductions from the New to Old World, but recent molecular analyses of both plastid and nuclear DNA sequences has shown the Old World species of spiny solanums are a monophyletic group [18], [27]. The *Solanum* species considered to be the closest wild relatives of the eggplant are all African [24]; this somewhat enigmatic pattern of a largely Asian crop with wild relatives in Africa has bedevilled investigation of eggplant origins and evolution. The early 20^th^ century German botanist Georg Bitter attempted a complete revision of the African solanums in his monumental, multi-volume *Solana Africana*; his treatment was based on relatively small samples compared to what is available today, and has proved difficult due to the destruction of many of the type specimens of taxa he described during the bombing of Berlin in the Second World War [28].

Eggplant taxonomy, evolution and biogeography were studied intensively by the late Richard Lester and his colleagues [29], [30], [31], [32]. The accessions they used for these studies were assembled by Lester and are now held in the germplasm collections of Radboud University in Nijmegen, The Netherlands (http://www.ru.nl/bgard/about_solanaceae/ru_solanaceae/) and at INRA in Avignon, France (http://w3.avignon.inra.fr/gafl/fr); these seventy accessions (and various subsets of them) were greenhouse grown and plants were not observed in the field in their native habitats. They proposed a variety of hypotheses to explain the complex pattern of wild, domesticated and semi-domesticated plants that form what has been called the “*S. incanum*-*S. melongena* complex” [27]. The work of Lester’s group using a variety of phenetic techniques is well-summarized in Daunay and Hazra [19] and so we will not repeat it here. Their work culminated in a classification for the group that recognised two species, each of which had a number of informal forms or groups [23], [29]; see Table 1 column 1. These “taxa” have been used to investigate crossing relationships [31] and are used in the EGGNET (EGGplant genetic resources NETwork) scheme for recording eggplant germplasm collections (see http://www.bgard.science.ru.nl/eggnet/eggnet01.html). The EGGNET descriptors and germplasm database tools are extensively used by germplasm curators and breeders [19].

**Table 1:** Equivalence between the classification of [23], [29] and the species recognised here^1^.

Informal taxa	Species recognised here	Distribution	Habitat
*Solanum incanum*group A	*Solanum campylacanthum* A.Rich.	Widespread in especially E Africa	Ruderal; many habitats
*Solanum incanum*group B	*Solanum campylacanthum* A.Rich	Southern narrow-leaved forms;southern Africa	Ruderal; many habitats
*Solanum incanum*group C	*Solanum incanum* L.	N Africa across Middle East to Pakistan	Deserts (drier than any of the other taxa)
*Solanum incanum*group D	*Solanum lichtensteinii* Willd.	Southern Africa	Ruderal; many habitats
*Solanum melongena*group E	*Solanum insanum* L.	Asia and Madagascar	Ruderal; many habitats
*Solanum melongena*group F	*Solanum insanum* L.	Easternmost form of *S. insanum*	Ruderal; many habitats
*Solanum melongena*group G	*Solanum melongena* L.	Southeast Asia	Cultivated, occasionally escaped; “primitive cultivars”
*Solanum melongena*group H	*Solanum melongena* L.	Cultivated worldwide	Cultivated; advanced cultivars
not treated	*Solanum aureitomentosum* Bitter	Higher elevations E Africa to Zambia	Ruderal
not treated	*Solanum cerasiferum* Dunal	Northern Africa, Senegal to Sudan	Ruderal
not treated	*Solanum linnaeanum* Hepper &P.M.L.Jaeger	South Africa/Mediterranean	Ruderal; many habitats
not treated	*Solanum rigidum* Lam.	Cape Verde Islands	Ruderal
not treated	*Solanum umtuma* Voronts. &S.Knapp	South Africa (KwaZulu-Natal)	Ruderal

1 The circumscription of the taxa is identical with the exception of the merging of “group A” and “group B” into *S. campylacanthum* and the addition of additional species not treated by [23], [29] now recognised by us and others [21], [27] as belonging to the eggplant clade.

One difficulty with these apparently informal taxa is that the informal designation can be truncated from a database taxon record (as happens in CGIAR’s GENESYS, https://www.integratedbreeding.net/genesys-global-portal-information-about-plant-genetic-resources-food-and-agriculture) thus potentially leading to considerable confusion as to identity of particular accessions (N. Castañeda, pers. comm.). Weese and Bohs [27] tested this classification and Lester’s hypothesis of eggplant migration patterns using DNA sequence data from Lester’s accessions and found that the A–G groups held up well, but that the South African species *S. linnaeanum* Hepper & P.-M.L.Jaeger was part of the monophyletic group comprising the eggplant and its relatives. Meyer et al. [21] used a much wider set of samples and came to similar conclusions, and additionally suggested that southeast Asian and Indian material were the same taxon.

Cultivated plants can be particularly challenging taxonomically, due not only to over-description using botanical criteria of entities that are entirely human-controlled [10] but also due to incomplete sterility barriers between them and their wild progenitors [5]. Most crop plants today are largely human constructs, and while derived from wild species by human selection, they are not currently under a natural selection regime and have not necessarily undergone speciation analogous to that occurring in wild taxa. For this reason, we prefer to designate cultivated plants as species distinct from their wild progenitors, as has been done for tomatoes by Peralta et al. [8] rather than subsuming the cultivar as a subspecies or variety of the wild progenitor as has been done for eggplants by some authors in the past [33]. The cultivated plant is not part of the same selection regime as its relatives growing in the wild even though interfertility, sometimes considerable, might be present; see [34], [35] for discussions of species concepts.

In this paper, we present a synopsis of the names and commonly used synonyms for the taxa we recognise for the eggplant and its close relatives so that these names and their equivalence to previous systems are widely available both inside and outside the taxonomic community. The identity and names for eggplant CWR is essential for collection and preservation of material for breeding in the face of environmental change and for the management of germplasm collections generally. This is also important in light of the controversy [19] surrounding the development of genetically-engineered “Bt brinjal” and the potential for confusion over the naming of the wild relatives in the context of assessing gene flow in the field.

## Materials and Methods

Our circumscription of the Eggplant clade follows Weese and Bohs [27] and also includes taxa that have been treated by other authors as being related to the eggplant [36], [37]; it is also based on our work with African solanums using DNA sequence data [38].

This paper is based on the taxonomic work done as part of a complete monographic treatment of the spiny solanums of continental Africa and Madagascar; species circumscription was based on analysis of herbarium specimens from throughout continental Africa and Madagascar, supplemented with material from the rest of the world where appropriate (e.g., Asia and the Cape Verde Islands). All specimens examined for these studies are recorded in the Solanaceae Source database and are available at http://www.solanaceaesource.org. The *Flora of Tropical East Africa* [25] treats four of these species in detail (*S. campylacanthum*, *S. incanum*, *S. lichtensteinii*, *S. melongena*) with complete synonymy and full descriptions, the rest are treated in our upcoming monograph of African solanums; a complete description of *S. rigidum* from the Cape Verde Islands is provided on Solanaceae Source.

We present only partial synonymy in the synopsis and list only names that have been used in previous studies of eggplant taxonomy and origins [19], [23], [29]. Many of the names we recognise as synonyms of the species here have been used at a variety of ranks; many of the synonyms of *S. campylacanthum* were first described at the species level by the German botanist Udo Dammer, then later recombined at the infraspecific level by Georg Bitter [36] in either *S. incanum*, *S. campylacanthum, S. panduriforme* or *S. delagoense*. Full synonymy and listings of the type specimens for all of the species treated here can be found on the Solanaceae Source website (http://www.solanaceaesource.org), all species except *S. melongena*, *S. rigidum* and *S. umtuma* in *Flora of Tropical East Africa* [25], and in our upcoming monograph. Complete synonymy of *S. melongena* is extensive and involves the description of many cultivars as botanical species (as was the case in the cultivated potato [10]); this is presented on Solanaceae Source and the complex typification issues will be dealt with in another publication.

## Results and Discussion

### Taxonomic Treatment

**Figure 1:**
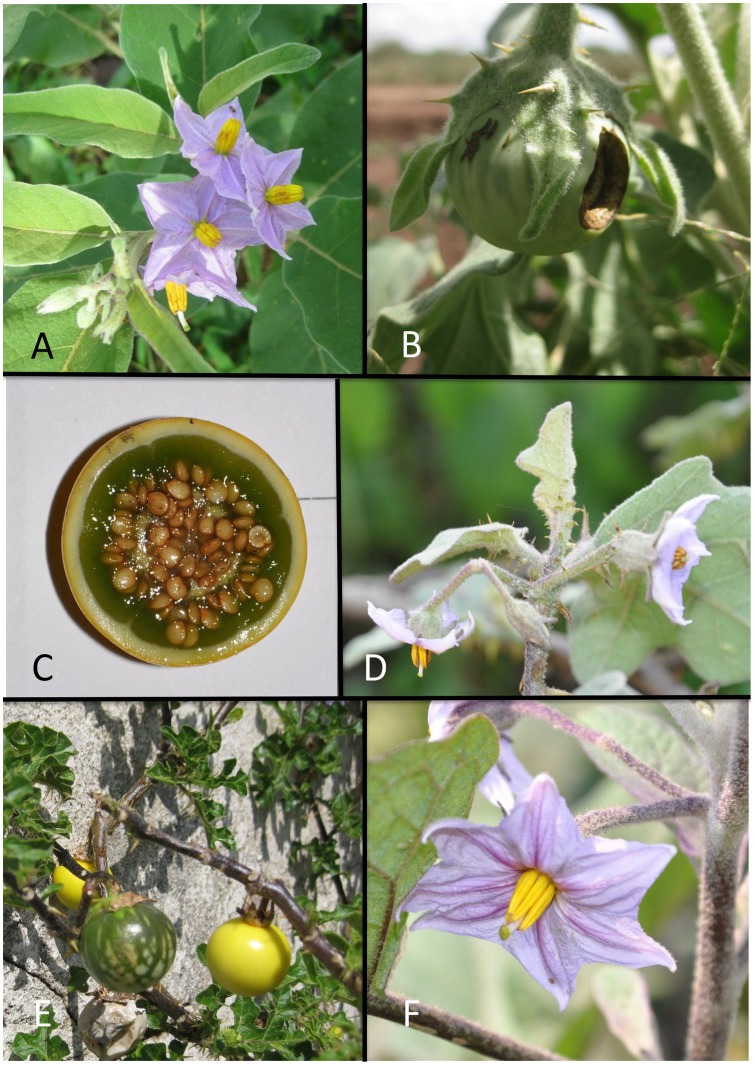
Representative flower and fruit morphology of eggplant and its wild relatives. A. *Solanum campylacanthum* inflorescences (Kenya - *Vorontsova et al. 157*); B. *Solanum incanum* immature fruit (Kenya – *Vorontsova et al. 203*); C. *Solanum insanum* fruit cross-section (China – *Wang et al. 2047*); D. *Solanum insanum* inflorescence with several hermaphrodite flowers (China – *Wang et al. 2039*); E. *Solanum linnaeanum* with yellow mature and mottled green immature fruit, note highly dissected leaves (Spain - *Knapp IM-10096*); F. *Solanum melongena* flower with duplicated parts (China – *Wang et al. 2042*). Photographs: A, B taken M.S. Vorontsova; C, D, E, F taken by S. Knapp.

The variability of species in the group, coupled with their very similar morphology means that a dichotomous key is difficult to use for identification. Comparison of key morphological characters is presented in Table 2, this should aid in identification of individual plants. Eggplants and their relatives are strongly andromonoecious, a derived breeding system characterised by a single or few long-styled, hermaphroditic flowers at the base of the inflorescence with more distal short-styled flowers that have a purely male function [39]. Many modern varieties of eggplant have been selected to bear a single long-styled flower to improve fruit size uniformity, as multi-fruited inflorescences tend to have variable fruit size. We have used the length and shape of the calyx lobes of long-styled flowers in the key and in Table 2; calyx lobes of short-styled flowers are often different in morphology and tend to be more similar across the species. Trichomes of vegetative parts in these plants are stellate with a star of lateral rays arranged in a single plane and a central, often elongate midpoint perpendicular to the rays or multangulate, with a similar stalk but with the rays more numerous and not in a single plane. These trichomes can be sessile or have a very short stalk (multicellular base) like those in *S. campylacanthum*, or the stalk can be elongate (e.g., *S. aureitomentosum*) giving the plants a woollier appearance. These characters can be seen with a 10× hand lens. All of these species have purple to pale lilac flowers (see Fig. 1A, D, F), with some white-flowered individuals occurring in individual populations.

**Table 2:** Key vegetative differences of eggplants and wild relatives.

Species	Leaf shape	Leaf base	Leaf lobe apex	Secondaryleaf lobing	Total calyx(lobe) length	Calyx lobe shape	Calyx lobe apex	Prickle # on calyx
*Solanum aureitomentosum*	Ovate	obtuse to cordate	rounded to obtuse	absent	12–19 (7–10) mm	ovate to oblong and leafy	obtuse	30–60
*Solanum campylacanthum*	ovate to elliptic orlanceolate	rounded to cordate	rounded, sometimes acute	absent	7–15 (5–10) mm	deltate to narrowly deltate	acute to obtuse or acuminate	0–20
*Solanum cerasiferum*	ovate to elliptic	attenuate	rounded to acute	sometimes present	7–12 (4–7) mm	deltate to narrowly deltate	acuminate	0–20
*Solanum incanum*	Ovate	rounded to cordate	rounded	absent	6–10 (2.5–5) mm	deltate to narrowly deltate	acute to obtuse	15–60
*Solanum insanum*	Ovate	truncate, sometimes obtuse	acute (the very tipssometimes rounded)	absent	5–10 (4–6) mm	deltate	acute	0–15
*Solanum lichtensteinii*	Ovate	cordate, sometimes cuneate	rounded	absent	7–15 (3.5–6) mm	deltate to narrowly deltate	acute to obtuse	20–50
*Solanum linnaeanum*	elliptic, sometimesovate or obovate	cuneate or obtuse	rounded	always present and oftenwell-developed	10–14 (5–6) mm	deltate to ovate	acute to rounded	30–100
*Solanum melongena*	Ovate	cordate to obtuse	rounded	absent	10–40 (5–17) mm	deltate to narrowly deltate	acute to long-acuminate	0(−30)
*Solanum rigidum*	elliptic	attenuate	acute	absent	10–12.5 (6–7) mm	narrowly triangular	acuminate	5–25
*Solanum umtuma*	elliptic	cuneate to truncate	obtuse to acute	often present	11–22 (7–10) mm	ovate and leafy	obtuse	30–80

### Key to the Species of the Eggplant Clade

1a. Fruit with soft pericarp, in a variety of shapes and colours, edible, the mesocarp spongy, usually white or cream, sometimes green or green-tinged; fasciation common, number of flower parts up to 8 and the ovary inflated. Cultivated.*S. melongena*
1b. Fruit spherical, yellow (sometimes pale orange-yellow) when mature, with comparatively hard pericarp, not palatable, the mesocarp usually green and jelly-like, if slightly spongy less than 1 cm thick; wild plants, flowers 5-merous.2
2a. Leaves deeply and ornately lobed with primary lobes extending 2/3–3/4 of the distance to the midvein and secondary lobes always present. Southern Africa and around the Mediterranean. *S. linnaeanum*
2b. Lobes entire or lobed, lobes extending up to 2/3 of the distance to the midvein, secondary lobes usually not present. 3
3a. Leaf lobes obtuse to acute at the tips, sometimes rounded, sometimes with small secondary lobes; lobes ¼-2/3 of the distance from the leaf outline to the midvein. Leaves and young stems glabrescent to moderately pubescent. 4
3b. Leaf lobes rounded at the tips, sometimes obtuse, never with secondary lobes; lobes up to 1/3(1/2) of the distance from the leaf outline to the midvein. Leaves and young stems usually densely pubescent (hairs overlapping). 6
4a. Calyx lobes on long-styled flowers 7–10 mm long, ovate and leafy, obtuse at the tips. South Africa.*S. umtuma*
4b. Calyx lobes on long styled flowers 4–7 mm long, deltate or long-triangular with acuminate tips. 5
5a. Calyx lobes on long-styled flowers 4–7 mm long, deltate, ca. 1/6 as long as the fruit at maturity. Continental Africa north of the Equator.*S. cerasiferum*
5b. Calyx lobes on long-styled flowers 6–7 mm long, long-triangular, 1/2 to 2/3 as long as the fruit at maturity. Cape Verde Islands.*S. rigidum*
6a. Stems prickly (but see Table 3). Prickles straight or slightly curved, usually with broad bases. Corolla on long-styled flowers 1.8–2.5 cm in diameter. Anthers ca. 4.5 mm long. Asia from the Philippines to southeast Asia, India and Madagascar.*S. insanum*

**Table 3:** Character suite for distinguishing ambiguous specimens of *S. insanum* and *S. melongena.*

Character	*Solanum insanum*	*Solanum melongena*
Stems	prickly	smooth
Leaf lobes	acute	obtuse
Leaf base	truncate or acute	cordate to obtuse
# of long-styled flowers	1–4	only 1
Flowers	strictly 5-merous	fasciated (with supernumerary parts)
Fruit size	1–3 cm in diameter and length	larger than 3 cm in diameter and/or longer than 3 cm in length
Fruit pulp	green and jelly-like	spongy

6b. Stems prickly or smooth. Prickles, if present, curved or straight. Corolla on long-styled flowers 2.5–4.5 cm in diameter. Anthers 5–9 mm long.7
7a. Leaves usually entire, sometimes lobed. Trichomes on the lower leaf surface sessile or with short stalks to only 0.1 mm long. Fruits 1.5–3 cm diameter. Ubiquitous weed at low altitudes in East Africa.*S. campylacanthum*
7b. Leaves lobed. Trichomes on the lower leaf surface with stalks to 0.5(1) mm long. Fruits 2.5–4.5 cm diameter.8
8a. Leaves velvety red-brown on the upper surface. Calyx lobes on long-styled flowers ovate to oblong, leafy, 7–10 mm long. Southern Africa.*S. aureitomentosum*
8b. Leaves yellow-green to green-brown on the upper surfaces. Calyx lobes on long-styled flowers deltate, 2.5–6 mm long.9
9a. Leaves concolorous to weakly discolorous, pubescence yellowish. Leaves ca. 1.5 times longer than wide. Young stems (dry specimens) terete to angular; NE Africa and the Middle East to Pakistan.*S. incanum*
9b. Leaves strongly discolorous, pubescence dirty greenish-brown on the upper surfaces and whitish on the lower surfaces. Leaves 1.5–2.5 times longer than wide. Young stems (dry specimens) with pronounced raised longitudinal ridges. Southern Africa.*S. lichtensteinii*

### Synopsis

1. Solanum aureitomentosum Bitter, Repert. Spec. Nov. Regni Veg. 11: 18. 1912.
2. *Solanum chrysotrichum* C.H.Wright, Kew Bull. 1894: 129. 1894, nom. illeg., later homonym of *Solanum chrysotrichum* Schltdl.

Distribution. Southern Africa, from Southern Democratic Republic of the Congo to Angola, southern Tanzania, Zambia, and Zimbabwe; roadsides, *Brachystegia* Benth. (Fabaceae, Caesalpinoidae) woodland and grassland; 800–1600 m elevation.

*Solanum aureitomentosum* is a distinctive densely woolly plant with leafy calyx lobes in long-styled flowers. It is very similar to and partly sympatric with *S. lichtensteinii* but we have chosen to recognise it due to the distinctness of the combination of morphological characters and its more high elevation forested habitat. Field studies of these (and all species of the group) at local scales will be useful in furthering the understanding of this variation. No accessions we identify as *S. aureitomentosum* have been used in previous studies of this group, nor can we find any evidence for accessions of this species in eggplant germplasm collections.

1. Solanum campylacanthum A.Rich, Tent. Fl. Abyss. 2: 102. 1850.
2. *Solanum bojeri* Dunal, Prodr. [A.P. de Candolle] 13(1): 344. 1852.
3. *Solanum panduriforme* Drège ex Dunal, Prodr. [A.P. de Candolle] 13(1): 370. 1852, as “*panduraeforme*”.
4. *Solanum delagoense* Dunal, Prodr. [A.P. de Candolle] 13(1): 349. 1852.

Distribution. Ubiquitous weed of low altitudes in Southern and Eastern Africa: roadsides, abandoned cultivation, savanna, bushland, dunes, forest edges etc.; usually 0–2000 m, but has been recorded up to 2300 m elevation.

**Figure 2:**
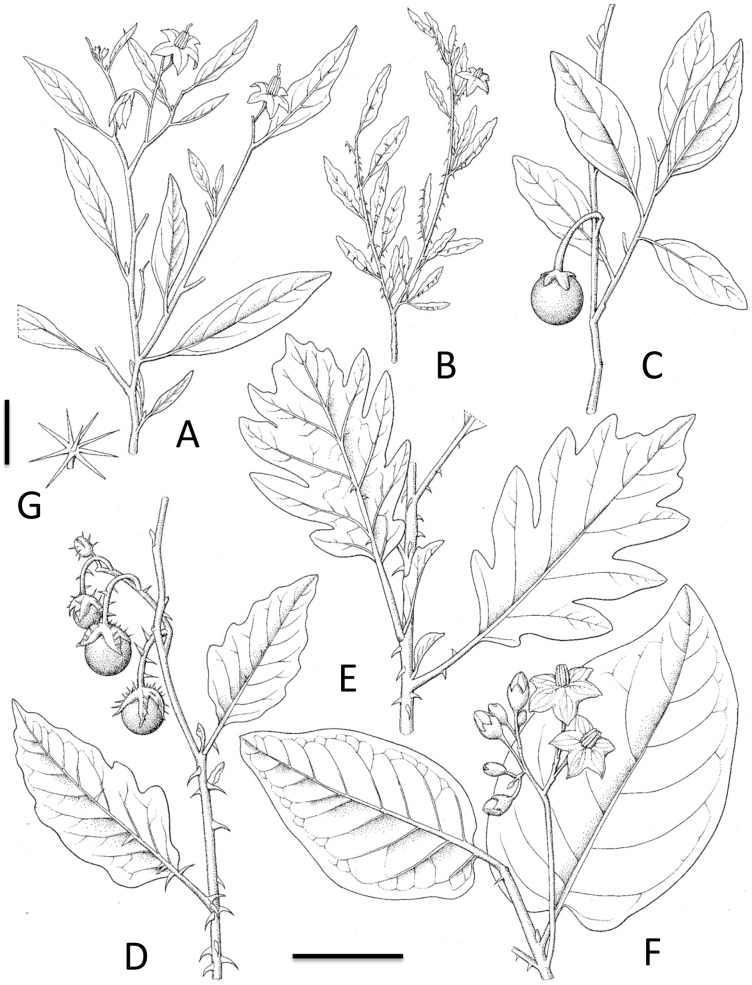
A sample of form and leaf variation in *Solanum campylacanthum* A.Rich. from across its morphological and geographical range showing the cline in leaf shape from south to north. A. Habit with narrow elliptic leaves. B. Habit with small leaves, small flowers, and dense curved prickles. C. Habit with ovate leaves and no prickles. D. Habit with large prickles and multiple fruits per infructescence. E. Habit with lobed leaves. F. Habit with cordate leaves. G. Short-stalked trichome from abaxial side of leaf. (Based on: A, *Mott 11B*, country; B, *Gilfillan 6056*, country; C, *Torre 7145*, Mozambique; D, *Stewart E33*, country; E, G, *Friis et al. 8107*, Ethiopia; F, *Friis 8505*, Ethiopia) Scale bar: A–F = 4 cm; G = 0.4 mm. Drawn by Lucy T. Smith.

*Solanum campylacanthum* is extremely widespread and variable (75 heterotypic synonyms [25]), particularly with respect to leaf morphology (Fig. 2) but flowers are relatively uniform throughout its range (Fig. 1A). The vast majority of wild egglant relatives collected in Africa belong to this species, which is commonly and incorrectly called “Solanum incanum” (see discussion of *S. incanum* below). Our concept of this species corresponds to “Solanum incanum group A” and “Solanum incanum group B” of Daunay et al. [23]; “group B” comprises those plants with narrower leaves from the southern part of the species distribution that have been recognised by some as *S. delagoense*, *S. panduriforme* or as infraspecific taxa based on those epithets [31]. From our examination of many herbarium specimens throughout Africa we conclude that this variation represents a north-south cline with leaf shape narrower in more southern populations. The variation is continuous and we do not think it warrants taxonomic recognition at either the specific or infraspecific level. *Solanum campylacanthum* can form dense stands of monomorphic plants through vegetative reproduction by underground stems; this can lead to the impression that variation is at a population rather than an individual level. Samuels [31] showed that “A” and “B” were fully interfertile, and thus classified them as subspecies.

Crossability of *S. campylacanthum* with other members of the group has proved difficult [40] and one-way pre-zygotic barriers have been suggested as the reason for this failure of fruit set in crosses with *S. incanum* and *S. lichtensteinii* [31]. It is possible that some of these difficulties could be due to ploidy differences within *S. campylacanthum*. Anaso and Uzo [41], [42] reported tetraploidy in *S. campylacanthum* from Nigeria (reported as *S. incanum*); their study illustrates the problems with inconsistent application of names in this group, they compared wild tetraploid “S. incanum” ( = *S. campylacanthum*) with cultivated diploid “S. incanum” ( = *S. aethiopicum* L., the unrelated scarlet eggplant). Fortunately they illustrated the plants used in the studies and identification of their material is clear even in the absence of vouchers. There has been an assumption that all relatives are, like the eggplant itself, diploid and earlier cytogenetic studies have not explicitly cited vouchers for counts of “S. incanum” so verification of identities of taxa counted is difficult. Ploidy level variation in *Solanum* is most common in the potatoes, where the cultivated potato has a number of ploidy forms and wild species vary from diploid to hexaploid [12], and in the Morelloid clade (*S. nigrum* L. and its relatives; see [43]), but it also occurs in some species of the Leptostemonum clade, particularly in widespread weedy species such as *S. elaeagnifolium* Cav. [44].

1. Solanum cerasiferum Dunal, Prodr. [A.P. de Candolle] 13(1): 365. 1852.

Distribution. From Senegal to Cameroon, Sudan and Ethiopia; continental Africa north of the Equator; fallow land, scrubland, and woodland, 450–1200 m elevation.

*Solanum cerasiferum* is morphologically very similar to and partly sympatric with *S. campylacanthum*, from which it differs in its lobed leaves with attentuate bases and sparser pubescence. It has a more northern and western distribution than *S. campylacanthum*, with some populations with intermediate morphological character combinations known from the northern part of the Democratic Republic of the Congo. West African populations with a dense cover of trichomes also resemble sympatric populations of *S. incanum*. Individual specimens from the area of sympatry can be difficult to identify, but a careful examination of leaves (particularly the bases) will enable differentation (see Table 2). *Solanum cerasiferum* usually has several long-styled flowers and fruits per inflorescence, and generally exhibits weaker andromonoecy than other African eggplant relatives. It is not known if *S. cerasiferum* forms clonal populations as does *S. campylacanthum*.

1. Solanum incanum L., Sp. Pl. 188. 1753.
2. *Solanum sanctum* L., Sp. Pl. ed 2: 269. 1762, nom. illeg. superfl.

Distribution. Ethiopia, Somalia, Arabia, and the Middle East to Pakistan, with some populations in N Kenya, Sudan, and extending to westwards to Mali; thickets, scrubland, and desert savanna; 0–1900 m.

Application of the name *S. incanum* has been incredibly confused and variable since its first description. This could be grounds for its rejection (see *S. linnaeanum* below), but we feel its common use to describe eggplant relatives merits its re-circumscription and careful re-use in a more restricted context than previously (e.g., [29]). The type specimen of *S. incanum* chosen by Hepper and Jaeger [45] matches material from the Middle East in being densely yellow pubescent with shallowly lobed leaves. Due in part to the misapplication of the name *S. incanum* to material from India and southeast Asia and confusion over the differences between *S. incanum* and *S. insanum*, North African specimens of *S. incanum* as defined here were often identified and sometimes named as varieties of *S. coagulans* Forssk., an unrelated North African species that can easily be distinguished from *S. incanum* by its fragrant zygomorphic flowers and berry enclosed in an accrescent calyx; see complete synonymy in [25]. The most common misapplication of the epithet “incanum” is its use to describe any wild eggplant relative from Africa, most commonly *S. campylacanthum*.

*Solanum incanum* (Fig. 1B) is a species of dry regions from northern Kenya to Pakistan and in general occurs in drier areas than do other species of the group, although all are weedy and occupy a wide variety of habitats. It is morphologically most similar to *S. lichtensteinii* of southern Africa and clustered with that species in phenetic analyses [29], [30]. The species can be easily distinguished by geography and by the young stems on dry specimens that are more deeply ridged in *S. lichtensteinii* and only shallowly or not at all ridged in *S. incanum*.

Lester and Hasan [29] proposed that their “S. incanum group C” ( = *S. incanum* as defined here) was the ancestral type and that all the rest of the species were derived from it in a bidirectional manner (i.e., *S. melongena* to the east and *S. campylacanthum* to the south, then giving rise to *S. lichtensteinii* still further to the south); if polyploidy is indeed occuring in this group (see above under *S. campylacanthum*) this scenario needs re-examination. Chromosome counts have not been published for material that is verifiably *S. incanum*, but high fertility in crosses with *S. melongena* [19] and molecular work with co-dominant SSR markers [46] suggests it is diploid. *Solanum incanum* is being used in eggplant breeding programmes as a source of variation for phenolics content and resistance to drought as well as to develop ILs (introgression lines, see [47]; http://zamir.sgn.cornell.edu/Qtl/il_story.htm) as a resource for eggplant breeding [46].

1. Solanum insanum L., Mant. 1: 46. 1767.
2. *Solanum undatum* Lamarck, Tabl. Encycl. 2: 22. 1794.
3. *Solanum cumingii* Dunal, Prodr. [A.P. de Candolle] 13(1): 359. 1852.

Distribution. India to SE Asia, also found in Madagascar and Mauritius; degraded scrubland and secondary vegetation, 0–500 m elevation.

Our circumscription of *S. insanum* comprises “S. melongena group E” and “S. melongena group F” of Daunay et al. [23]; this includes those plants considered by them to represent wild progenitors and straggling prostrate forms they considered to be feral “reversions” from cultivated forms. This variation in habit is found in some other species of *Solanum* such as *S. arcanum* Peralta, a wild tomato from northern Peru [48]. Populations of *S. insanum* we have seen in southern China often have a mixture of forms; prostrate forms appear to grow in more open areas. Common garden experiments coupled with inter-lifeform crosses are necessary to determine the basis of this habit difference.

*Solanum insanum* is almost certainly the wild progenitor of the cultivated eggplant and is fully interfertile with it [19]. Meyer et al. [21] used AFLPs to test the relationships of wild and cultivated landrace eggplants and suggested that all Asian plants they analysed represented a single species (their *S. incanum*+*S. undatum* = our *S. insanum*) that was possibly of hybrid origin or had crossed repeatedly with local landraces of *S. melongena*. Regardless of which of these two scenarios is the case, *S. melongena* is likely to have had its origin(s) from amongst populations of Asian *S. insanum*, as Meyer et al. [21] pointed out. *Solanum insanum* is used medicinally in south China and is considered distinct from the cultivated *S. melongena* by local people (pers. obs.). Because *S. insanum* and cultivated *S. melongena* are highly interfertile and repeated introgression occurred and is still occurring between wild and cultivated plants individual plants can sometimes be difficult to assign to species unambiguously. In this case we have adapted the method used by Peralta et al. [8] for naming plants that were morphologically intermediate between the cultivated tomato (*S. lycopersicum*) and its wild progenitor (*S. pimpinellifolium* L.). They [8] defined a suite of characters that distinguish each species and an individual specimen having a majority of one set of these is called that species. Table 3 lists the suite of characters we have used for the eggplants; for example, a specimen with several long-styled, 5-merous flowers (Fig. 1D), non-prickly stems and juicy green fruit pulp (Fig. 1C) would be called *S. insanum*, while a specimen with prickly stems, rounded leaf lobes, one long-styled fascinated flower (Fig. 1F) and large fruit would be called *S. melongena*. Fruit colour is not a particularly reliable character, as it changes through fruit development (see Fig. 1E); *S. melongena* fruits are eaten when they are unripe, so the various green and purple fruits eventually become yellow or brownish yellow if left to ripen completely.

Hepper and Jaeger [45] clearly describe how Linnaeus described *S. insanum* as distinct from his earlier *S. melongena* by citing its prickly stems (in addition to calyx) thus indicating he considered *S. insanum* a new species and not a replacement name for *S. melongena* as *S. sanctum* was for *S. incanum* in [49]. It has been suggested that *S. insanum* was a misprint for *S. incanum* [45] but although it is unfortunate the two names are very similar they are not considered confusable (R. Brummitt, pers. comm.). Meyer et al. [21] erroneously considered *S. insanum* as illegitimate.

1. Solanum lichtensteiniiWilld., Enum. Pl. (Willdenow) 238. 1809.

Distribution. From South Africa to Angola, DR Congo, and Tanzania; dry grassland, woodland, and thickets; 500–2000 m.

*Solanum lichtenstenii* is morphologically similar to *S. incanum* in being densely pubescent with long-stalked trichomes, but can be distinguished from it geographically and by its ridged young stems. In herbarium specimens *S. lichtensteinii* plants have a greyer tone than the yellowish green *S. incanum*, but this character is difficult to quantify. Individuals of *S. lichtensteinii* in upland dry areas of South Africa can be of very small stature, with reduced entire leaves, while *S. incanum* is more consistent in plant size (shrubs of ca. 1 m) and no dwarf forms are known. Lester and Hasan [29] suggested that *S. lichtensteinii* (their “S. incanum group D”) had arisen from within “S. incanum group A” ( = *S. campylacanthum*) and that *S. incanum* in the strict sense arose from northern populations of *S. lichtensteinii*. Phylogenetic results [27] show this not to be the case, *S. lichtensteinii* is sister to the South Africa *S. linnaeaneum* and *S. campylacanthum* is sister to the rest of taxa sampled; this pattern of relationships is confirmed with a larger data set including more species of African solanums [38] where *S. umtuma* is also part of a clade including *S. lichtensteinii* and *S. linnaeanum*.

1. Solanum linnaeanum Hepper & P.-M.L.Jaeger, Kew Bull. 41: 435. 1986.

Distribution. Probably native to South Africa and naturalised around the Mediterranean in disturbed, often coastal, habitats worldwide; sand dunes, grass, forest margins, river banks, and roadsides at 0–1200 m elevation.

*Solanum linnaeanum* has long been referred to has *Solanum sodomaeum* L. or *Solanum hermannii* Dunal, the latter name is illegitimate and the former has been rejected according to the rules of botanical nomenclature and can therefore not be used [50], [51]. This species is probably native to South Africa and has been introduced into the Mediterranean region where it is now common. *Solanum linnaeanum* is morphologically quite distinct from the rest of the eggplant wild relatives with its deeply incised, almost glabrous leaves (Fig. 1E). In Spain, fruits of *S. linnaeanum* do not appear to be eaten by any animals, it is common to find fruits from several years still on the plant. The relationship of *S. linnaeanum* to the eggplant wild relatives was first clearly shown by Weese and Bohs [27]. It was previously used as the female parent in the creation of the first linkage map for eggplants [52] despite the cross only proving possible through embryo rescue (M.-C. Daunay, pers. comm.). The accession used by Weese and Bohs [27] was Mediterranean in origin, so the relationship of *S. linnaeanum* with the other South African species *S. lichtensteinii* is intriguing and perhaps indicative that it is introduced in the Mediterranean. *Solanum linnaeanum* has been used in analyses of resistance to important diseases such as bacterial wilt [53] and, similarly to *S. incanum*, is a candidate for the creation of ILs that would be valuable resources for eggplant breeding (M.-C. Daunay, pers. comm.).

1. Solanum melongena L., Sp. Pl. 186. 1753.
2. *Melongena ovata* Mill., Gard. Dict., ed. 8. Melongena no. 1. 1768.
3. *Melongena tereta* Mill., Gard. Dict., ed. 8. Melongena no. 2. 1768.
4. *Melongena incurva* Mill., Gard. Dict., ed. 8. Melongena no. 3. 1768.
5. *Melongena spinosa* Mill., Gard. Dict., ed. 8. Melongena no. 4. 1768.
6. *Solanum album* Lour., Fl. Cochinch. 129. 1790.
7. *Solanum ovigerum* Dunal, Nat. Hist. Solanum 210. 1813.

Distribution. Cultivated worldwide throughout the tropics and subtropics outside and in the temperate zone in the summer or in glasshouses. The greatest diversity of landraces and cultivars is found in Asia (India, China and southeast Asia), with secondary centres in the Middle East and around the Mediterranean.

**Figure 3:**
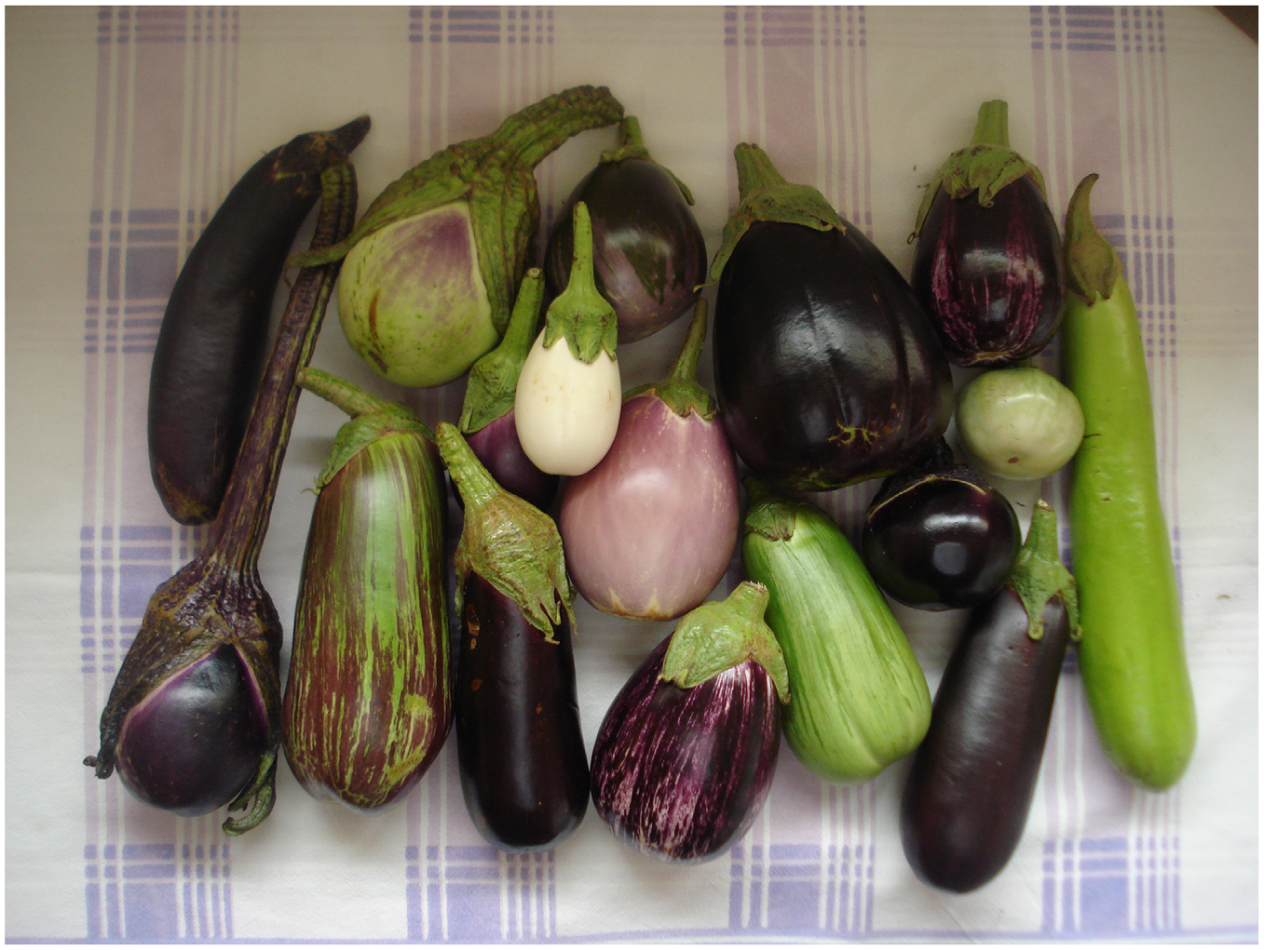
Fruit shape and colour variation in cultivars of the common eggplant, *Solanum melongena*. Photograph taken by J. Prohens.

We recognise as *S. melongena* only cultivated plants, including both “primitive” (“S. melongena group G”) and advanced cultivars (see Table 1). The 18^th^ century botanist and gardener Philip Miller [54] recognised the eggplant as its own genus based on its “one-celled” fruit, as he did the tomato based on its “many-celled” fruit, and castigated Linnaeus for sinking both into what he considered the overlarge genus *Solanum*. He described several “species” of his genus *Melongena* differentiated by their fruit shape and colour; these are certainly cultivars and would not be named as species today (Fig. 3 illustrates a sample of the diversity of eggplant fruit shapes and colours). *Solanum melongena* was probably domesticated multiple times [21] from populations of *S. insanum* and considerable gene flow still occurs between the two species. *Solanum insanum* is a wild plant, although often growing in disturbed areas including those around villages, while *S. melongena*, even the most primitive cultivars, is always in close association with people. Using the suite of characters in Table 3 will help with identifying difficult specimens (see above under *S. insanum*).

Flowers of *S. melongena* are often fasciated and have more than the standard 5 parts (Fig. 1F), this is caused by meristem mutations increasing the number of floral organs in the whorl [55]; the increased number of complicated carpels and the spongy mesocarp may have been what led Miller [54] to think the fruit had only a single locule. Domestication trends in *S. melongena* have involved size, shape and taste [56], [57] and the diversity of landraces in the area of origin is very large. Using a combination of historical, morphological and molecular information, Meyer et al. [21] suggested that *S. melongena* had been domesticated at least twice, and that it had been brought from east to west into Europe from India by Arab traders and from China east to Japan. Their analyses suggest that a cluster of landraces they called “S. melongena subsp. ovigerum” (a combination not validly published according to the Code [51]) from Malaysia represented a third domestication event; more population-level sampling and markers may reveal the origins of this pattern. The type of *S. ovigerum* (the basis for “S. melongena subsp. ovigerum”) is not from Malysia, but instead was a cultivated plant with purple or white ovate-oblong (egg-shaped) fruit. Nonetheless, the distinctiness of these southeast Asian genotypes is intriguing and merits further investigation. Hurtado et al. [58] used genomic SSRs together with morphological passport data to examine variation in cultivated germplasm of *S. melongena* landraces and advanced cultivars from China, Spain and Sri Lanka. Different selection pressures appear have been applied in the different regions, leading to typical local trait combinations, and they suggest that germplasm collections should also take care to include extensive samples from centres of cultivated diversity as well as wild species.

The nomenclature and synonymy of *S. melongena* is complex, and is complicated by the description of many plants grown in European botanic gardens with slightly different fruit morphologies as distinct species, type specimens of these names with only flowering material preserved if at all, and that many of these names were not published correctly according the rules of nomenclature [51]. The scientific name for cultivated eggplant has been *S. melongena* with consistent usage since the late 19^th^ century, thus little confusion exists over the identities of more derived cultivars. Landraces and cultivars are better named using the *International Code of Nomenclature for Cultivated Plants* [59] than by giving them botanical species and infraspecies names.

1. Solanum rigidum Lam., Tabl. Encycl. 2: 23. 1794.
2. *Solanum latifolium* Poir., Encycl. (Lamarck) 4: 303. 1797.

Distribution. Endemic to the Cape Verde Islands; a few old collections known from Barbados and Antigua were probably introduced via ships carrying enslaved Africans from the Cape Verdes to the Caribbean; found along washes and at roadsides, sea level to 1000 m elevation.

This species has long been known as *S. fuscatum* L., and was thought to be an American species introduced to the Cape Verde Islands [60]. *Solanum fuscatum* is a name of uncertain application [61], [62]; it has no type specimen and has recently been proposed for rejection [63] under the rules of the Code [51]. Morphology and molecular evidence (S. Stern and M.S. Vorontsova, unpublished) both show this species is a member of the eggplant clade and not an introduction from the Americas; it is endemic to the Cape Verdes and thus, despite its somewhat weedy nature, of conservation interest. The assumption that it was an American (New World) species has meant it has been treated as an invasive, rather than the endemic that it is. Its relationships to other eggplant relatives have not yet been rigorously assessed. *Solanum rigidum* resembles *S cerasiferum* morphologically but can be distinguished from it and other members of the group by its long triangular calyx lobes on long-styled flowers that are reflexed at the tips in fruit and the attenuate leaf bases.

1. Solanum umtuma Voronts. & S.Knapp, PhytoKeys 8: 4. 2012.

Distribution. Endemic to the province of KwaZulu-Natal in South Africa; occasional on grassland, scrub, and forest edges, on sandy soil, 50–1300 m elevation.

*Solanum umtuma* is sympatric with and closely related [38] to *S. linnaeanum*. *Solanum linnaeanum* has distinctive deeply incised leaves with rounded lobes (see Fig. 1E) while *S. umtuma* has more shallowly lobed leaves with acute lobes although some specimens have been seen with rounded lobes. The flowers of *S. umtuma* are usually pale lilac or white, while those of *S. linnaeanum* are purple. *Solanum cerasiferum* is also similar, but has less prickly calyces and deltate, rather than leafy, calyx lobes.

### Conclusions

We provide here names at the species level for eggplant relatives previously treated in an informal classification. Our treatment is based on examination of herbarium specimens from throughout the species ranges and takes into account natural variation, some of which is extremely great. We hope this will facilitate information exchange through databases and the future collecting of wild species for use in crop improvement. It is apparent that some ploidy level differences exist either between or within species, so chromosome counts and/or flow cytometry DNA content measures are needed for all eggplant wild relatives. Also, molecular work using highly repeatable co-dominant markers like SSRs and SNPs can complement morphological and chromosome cytology studies in order to understand the relationships between species as well as the genetic structure of populations within species. The reliance on a limited number of accessions, some of which lack exact provenance data, for much of the work done on eggplant origins and evolution means that new field collections with accurate provenance and good field observations at both a population and individual level are a priority for improved eggplant breeding in the future. The information provided here will be of great relevance for the management of genetic resources in germplasm collections as well as for the utilization of eggplant CWR by plant breeders, in particular those facing the challenges posed by enhanced biotic and abiotic stresses resulting from future environmental change.
